# Determinants of implementing of pet robots in nursing homes for dementia care

**DOI:** 10.1186/s12877-022-03150-z

**Published:** 2022-05-27

**Authors:** Wei Qi Koh, Elaine Toomey, Aisling Flynn, Dympna Casey

**Affiliations:** 1 National University of Ireland Galway, Galway, H91 E3YV Ireland; 2 University of Limerick, Limerick, V94 T9PX Ireland

**Keywords:** Dementia, Implementation, Barriers, Facilitators, Social robots, Pet robots, Robotic pets, Nursing homes, Long term care, Residential care, Care homes

## Abstract

**Background:**

Pet robots have been employed as viable substitutes to pet therapy in nursing homes. Despite their potential to enhance the psychosocial health of residents with dementia, there is a lack of studies that have investigated determinants of implementing pet robots in real-world practice. This study aims to explore the determinants of implementing pet robots for dementia care in nursing homes, from the perspectives of healthcare professionals and organisational leaders.

**Methods:**

A descriptive qualitative study, conceptualised and guided using the Consolidated Framework of Implementation Research (CFIR), was conducted. We conducted semi-structured interviews with healthcare professionals and organisational leaders from nursing homes. Data was transcribed and analysed using Framework Analysis, based on the CFIR as an a priori framework.

**Results:**

A total of 22 participants from eight nursing homes were included. Determinants were mapped to constructs from all five CFIR domains. Determinants relating to the characteristics of pet robots include their design, realisticness and interactivity, affordability, cleanability, perceived evidence strength and comparative advantages to live pets. Determinants relating to external influences (outer setting) include national regulatory guidelines, funding and networks with other organisations. With regards to characteristics of nursing homes (inner setting), determinants include the relevance of pet robots in relation to the needs of residents with dementia, alignment with care processes, infection control mandates and their relative priority. In the domain ‘characteristics of individuals’, determinants were associated with individuals’ beliefs on the role of technology, desires to enhance residents’ quality of life, and differential attitudes on the use of robots. Finally, in the domain ‘implementation process’, assessments and care planning were identified as determinants.

**Conclusions:**

Overall, while sentiments around determinants within CFIR domains of pet robots’ characteristics, outer setting and implementation process were similar, participants’ opinions on the determinants within the ‘inner setting’ and ‘characteristics of individuals’ were more varied. This could be due to different organisational structures, disciplinary differences and personal experiences of using pet robots. Many determinants in different domains were interrelated. Findings provide a springboard for identifying and designing implementation strategies to guide the translation of pet robots from research into real-world practice.

**Supplementary Information:**

The online version contains supplementary material available at 10.1186/s12877-022-03150-z.

## Background

Pet robots are technology-based substitutes for animal-assisted therapy. Animal-assisted therapy have demonstrated positive benefits on the psychosocial wellbeing of people with dementia, such as reducing depression, providing companionship and addressing unmet needs [1]. However, using live animals can be challenging due to issues such as logistical difficulties or potential transmission of zoonotic diseases [2]. Correspondingly, pet robots are considered as alternative solutions to circumvent such challenges, and have been used as non-pharmacological interventions to support the psychosocial health of people living with dementia [3]. There are several pet robots designed with varying levels of familiarity, realisticness and interactivity. Among the different robots, PARO is the most well-researched. While PARO was designed realistically to resemble a seal, it was intentionally designed as an unfamiliar animal to enhance its acceptability, based on developers’ notions that people would have fewer preconceptions or expectations of it [4]. Other examples of pet robots include the AIBO dog, Pleo (dinosaur) and the Joy for All (JfA) cat—some studies have suggested that older adults and people with dementia prefer familiarly designed pets such as cats and dogs [5]. Overall, current research suggests that realistically designed pet robots with fur-covering, such as PARO and the JfA cat, can evoke affective behaviours and are preferred by older adults and people with dementia [6, 7].

Three systematic reviews were conducted to evaluate the effectiveness and impacts of pet robots. While only one had a specific focus on using pet robots with people with dementia [8], most studies included in the other reviews focused on users who had mild cognitive impairment or dementia [9, 10]. Most studies included in all reviews were also focused on using PARO in long-term care. In terms of effectiveness and impacts, Leng and colleagues (2019) found a statistically significant reduction of behavioural and psychological symptoms of dementia (BPSD). Pu and colleagues (2019) had similar findings—using social robots including PARO decreased agitation, anxiety, medication use and loneliness. However, these effects did not reach statistical significance, possibly due to small samples and intervention heterogeneity. Similarly, while Abbott et al. (2019) did not find statistically significant reductions in agitation in their mixed method review, their qualitative synthesis demonstrated positive impacts of stimulating engagement, social interactions and mood amongst older adults and older people with dementia.

Although most included studies focused on the use of PARO, some researchers have argued that PARO has been overly researcher driven and technology driven, with insufficient consideration of real-world needs [11], which could explain low uptake in real-world practice [12]. For instance, a scoping review which synthesised the barriers to using PARO in care settings highlighted pragmatic issues such as cost [11]. The JfA cat represents a lower-cost alternative, and have been chosen by older adults in care homes as their preferred pet robot design among seven other alternatives [6, 13]. While the number of studies conducted to investigate the impacts of the JfA cat [14, 15] are significantly less than that of PARO, findings of their positive impacts on the psychosocial health of users resonate with previous studies. Hence, despite the need for more definitive evidence on the effectiveness and impact of pet robots, there is promising findings of benefits to the psychosocial health of nursing home residents with dementia.

The traditional research sequence often involves evaluating the efficacy and effectiveness of an intervention before knowledge of its implementation is being investigated [16, 17]. However, such step-wise approaches have been argued to have caused time lags between research discovery and uptake [16, 17]. As such, it is necessary to pursue knowledge on the implementation of pet robots alongside further investigation into their effectiveness, to improve the speed of knowledge creation in guiding the translation of pet robots from research into practice. This should entail a thorough understanding of implementation determinants [18]. A scoping review was conducted to synthesise findings from 53 studies, to identify barriers and facilitators to implementing social robots (including pet robots) for older adults and older people with dementia [19]. The review found that current research have been disproportionately focused on identifying determinants relating to the characteristics of robots, with a lack of studies investigating multilevel contextual determinants that can influence implementation, such as organisational workflows [19]. As such, the purpose of this study is to explore multilevel determinants to implementing pet robots in nursing homes for dementia care, from the perspectives of healthcare professionals and organisational leaders. The pet robots we focused on were PARO and the JfA cat, due to their realistic designs and the existing evidence-base suggesting their potential to positively impact the psychosocial health of nursing home residents with dementia.

To guide the comprehensive exploration of implementation determinants, we used the Consolidated Framework for Implementation Research (CFIR) as a guiding framework to undertake a comprehensive exploration of implementation determinants [20]. The CFIR is a meta-theoretical determinant framework that was conceptualised following a review and synthesis of theories of organisational change, dissemination, innovation, implementation, research uptake and knowledge translation [20]. Within this framework, 39 constructs that influence implementation are organised into five domains: 1) intervention characteristics, 2) inner setting, 3) outer setting, 4) individuals’ characteristics and 5) implementation process (Table 1).

**Table 1:** CFIR Domains

CFIR Domain	Description
Intervention Characteristics (i.e., characteristics of pet robots)	Refers to key characteristics of pet robots, such as complexity, design quality and packaging and cost
Outer Setting	Refers to external influences on the implementing organisation, such as external policies and guidelines
Inner Setting	Refers to the features of the implementing organisation (i.e., nursing home), such as residents’ needs and resources, readiness for implementation and implementation climate
Individuals’ characteristics	Refers to the characteristics of individuals (e.g., healthcare professionals) who are involved in implementation
Process	Refers to strategies for implementing pet robots, such as planning and engaging stakeholders

## Methods

### Study design and setting

A descriptive qualitative study [21, 22] was conducted. In a qualitative descriptive approach, researchers aim to stay close to the ‘surface of data and events’ [23] to explore and describe the phenomena of interest from the participants’ points of view. It also allows for flexibility in using a theoretical framework to guide the process of inquiry [23]. As such, this was chosen as the most suitable approach for our study. This study received approval from the National University of Ireland Galway research ethics committee (REF. 2020.10.014). Informed consent was obtained from all participants prior the study. Full details of the methods for this study are described in detail in a published protocol [24] and any deviations are clearly detailed below.

### Sampling and Recruitment

Purposive sampling was used to identify and recruit healthcare professionals (HCPs) with experience of providing care to residents with dementia, and organisational leaders (OLs) with experience of managing or leading a nursing home that provided care for residents with dementia. Sample size determination for this study was based on considerations from Sim and colleagues’ [25] outline of the numerical guideline and the conceptual model approaches. The former refers to suggestions based on recommendations from previous empirical studies, and the latter refers to sample size estimation based on information power [26] – this has been posited as a useful alternative to ‘data saturation determination’ [27]. Previous authors who used a theory based approach to qualitative inquiry have recommended an initial sample size of 10 participants [28]. Some considerations about information power included the non-specificity of the study objectives (due to heterogeneity of stakeholder groups) and quality of dialogue with participants, based on the lead researcher’s (WQK) experience with qualitative interviewing [29]. Based on these considerations, we anticipated an initial sample of at least 10 participants per stakeholder group. This decision was an iterative process, subjected to change based on informational power from the qualitative data was collected and analysed [26, 30]. To recruit study sites for participant recruitment, we leveraged on data from the Irish national open data portal and identified 33 nursing homes in a county in the west of Ireland that provided care for residents with dementia. WQK systematically contacted the nursing homes to explain about the study and invite them to participate. Eligible participants (Table 2) from nursing homes that agreed to participate were invited to join the study. Although we planned to recruit 2–3 HCPs and 2–3 OLs from four nursing homes in the West of Ireland, we had to extend recruitment to include additional nursing homes, due to difficulty recruiting sufficient participants from each organisation.

**Table 2:** Eligibility Criteria

Participants	Eligibility criteria
Healthcare professionals	•Provide direct care provision for nursing home residents with dementia •Can speak and understand English
Organisational leaders	•Has experience as a manager or leader in a nursing home, or has managed or led a team of care workers or organisational processes within the facility •Can speak and understand English

Twenty two participants from eight nursing homes participated in this study. Of seven invited organisations, six agreed to participate. Of 19 invited participants, all but one agreed to be interviewed. Three participants from two additional homes were recruited through snowball sampling. Table 3 shows the characteristics of the nursing homes. We anticipated that nursing homes would not have pet robots. However, two had the JfA cats, and one had both the JfA dog and cat. None had experience using PARO, except for one participant who used it during a trial approximately ten years ago. Participants comprised of 10 OLs and 12 HCPs (due to the heterogeneity of HCPs being included). A summary of their demographics can be found in Table 4.

**Table 3:** Characteristics of nursing homes

	**Sample size (n)**
**Total no. of nursing homes**	**8**
HSE-funded (i.e., public)	5
Privately funded	3
**Has a pet robot/pet robots**	
Yes	**3**
No	**5**
**Total number of residents**	
21 – 30	3
30 – 40	1
40 – 50	2
80—90	1
**Proportion of residents with a diagnosis of, or symptoms of dementia**	
20 – 30%	2
40 – 50%	1
50 – 60%	1
Over 80%	2
No information	2

**Table 4:** Characteristics of participants

	**Sample size (n)**
**Organisational leaders**	**10**
Assistant director/Director of nursing	6
Clinical nurse manager	3
Occupational therapy manager	1
***Healthcare professionals***	**12**
Nurse	5
Healthcare assistant	1
Activity coordinator	2
Occupational therapist	3
Physical therapist (Physiotherapist)	1
**Gender**	
Male	4
Female	18
**Age group (in years)**	
20 – 29	2
30 – 39	3
40 – 49	8
50 – 59	3
70 and over	1
**Years of experience in dementia care**	
1 to 3 years	3
4 to 6 years	3
7 to 9 years	3
Over 10 years	11
**Owns or has owned a pet**	
Yes	16
No	6
**Considers self as an animal lover**	
Yes	19
No	1
Unsure	2
Have seen a pet robot	
Yes	13
No	9
**Have used a pet robot**	
Yes	7
No	15

### Deviation from protocol

We also intended to recruit community-dwelling people with dementia, however this was not possible—this may be because participation would require them to think ahead about care provision in nursing homes, a future that may be difficult for them to contemplate, or due to challenges with executive cognitive functioning which may influence their ability to consider prospectively [31]. Furthermore, the study involved questions relating to organisational contexts within nursing homes, which may be difficult for community-dwelling people with dementia to discuss. Therefore in deviation from our protocol, we could not include community-dwelling people with dementia. However, to ensure that their viewpoints on implementing pet robots in nursing homes were considered, we consulted with an advisor with dementia from the Dementia Research Advisory Team [32] during the study conceptualisation and data collection as a part of Patient and Public Involvement (PPI) – this refers to the partnership with patients and the public in research, rather than ‘doing research for them’ [33]. The Dementia Research Advisory Team is comprised of people living with dementia and their carers who collaborate or provide advice in dementia research in Ireland [32]. A summary of the agenda for the PPI consultation sessions can be found in Additional File 1.

### Data collection

Data collection took place between August to November 2021. Participants were first introduced to the pet robots through a 5-min video, where the lead researcher (WQK) demonstrated their features and functions (Additional File 2). In-depth, semi-structured interviews were conducted by WQK subsequently, and each interview lasted between 31 to 54 min. The interview guide (Additional File 3) used to guide data collection was developed using domains and constructs in the CFIR [20] and findings from our preceding scoping review [19]. For instance, we placed emphasis on understanding organisation-related factors, which were identified as knowledge gaps that were not explicitly investigated in previous studies. These questions were piloted prior to data collection. All interviews were audio recorded. Due to the ongoing Covid-19 pandemic, we planned to conduct interviews primarily via Zoom or via the telephone, to minimise the risk of infection transmission through physical meetings. However, the option of physical (in-person) interviews was also offered to participants if preferred. The latter option depended on prevailing public health guidelines, which determined the practicability and safety of physical access into nursing homes. Fourteen interviews were conducted in-person at each participant’s nursing home, and 8 were conducted through videoconferencing via Zoom.

### Data analysis

Framework analysis was used to analyse the data [34, 35], using a combination of deductive and inductive approaches. First, all audio recordings were transcribed verbatim and uploaded onto NVivo12. The first ten transcripts were transcribed by WQK and the remaining transcripts were transcribed with professional transcription services. In step two, WQK and AF familiarised themselves with the data by listening and immersing in the interview transcripts and audio-recordings, keeping notes of any initial impressions, thoughts and ideas in relation to the CFIR, to remain attuned to emerging data whilst using CFIR as a starting point. Based on the initial notes from the first five interviews, we developed subcodes within the constructs and domains in the CFIR, and this constituted our preliminary framework. The third step involved identifying a framework that could be applied to the rest of the data through an iterative process of piloting our preliminary framework, to ensure that we remained attuned to emerging data. WQK and AF independently coded one interview, met up regularly to discuss any difficulties in applying the framework, and revised the framework categories to ensure that we remained attuned to any emerging data. After piloting the preliminary framework on five interviews, we developed a framework (Additional File 4) for the fourth step of indexing. In this step, WQK applied the framework to the rest of the transcripts. Next, all indexed data were charted onto a framework matrix by summarising participants’ interviews and arranging them by categories (i.e., CFIR constructs and subcodes). This facilitated analysis within and between each interview, and the preparation of data for mapping and interpretation. WQK reviewed the charted data to identify characteristics, differences and patterns in the data, and annotated impressions during this process. Attention was also paid to comparing the patterns of data between participants with and without experiences of using pet robots. The findings and interpretation were presented to AF and our PPI member, who were invited to provide feedback and suggest changes to the interpretation. These steps were not linear, and involved a reflective, analytical (iterative) process of moving forward and back between steps. For example, although the process of ‘identifying a framework” (step 3) was intended to precede “indexing” (step 4), the development of our framework was an ongoing process in our study to accommodate new subcodes that were created to capture the descriptions of data that did not fit in the existing framework during indexing. In addition, descriptions of some subcodes were revised. During the ‘mapping and interpretation’ process, we also moved back and forth to refer to the original transcripts to better understand and confirm patterns of data. This ensured that the data analysis remained a thoughtful and reflective process rather than being mechanistic, especially during the ‘indexing’ stage [34]. The Standards for Reporting Qualitative Research [36] was used to report the findings (Additional file 5).

### Findings

#### Domain 1: Characteristics of Pet Robots

This domain describes determinants relating to the characteristics of robots, such as their design, cost and evidence. Participants described them as being realistic, which they felt was important for acceptability and to not be considered infantilising. While PARO’s design as a seal was culturally unfamiliar, the JfA cat’s design as a familiar animal was thought to be more relevant and impactful. Some felt that PARO’s advanced interactive capacities were beneficial, however others doubted their essentiality, especially if they increased cost: *“Maybe people are just as happy if they feel it responds to them [HCP10]”.* Furthermore, some felt these features, such as PARO’s voice recognition abilities, might be restricted in a nursing home environment where noise levels are often high. Their robustness was also of concern, as residents with dementia may not understand how to care for the robot as a technical device: *“when you give such a pet to somebody with dementia, they have no concept of not holding it too tight or restricting its movement. It's very likely that they will, so I would be concerned about their durability [HCP4]"*. While their fur-covering contributed to appeal, their cleanability was a concern. This led a nursing home to dispose of a JfA cat during Covid-19. Most participants were unanimous that PARO’s cost was prohibitive, and that it would be unaffordable for their nursing homes. Organisations with the JfA cat learned about and acquired it through a central website for medical supplies, describing it as being more affordable.

Participants shared personal anecdotes of their experiences as supporting evidence for pet robots, which facilitated implementation: *“He’s so much happier. I think everybody would probably say that they see such a difference [HCP5]”*. Whilst not all had experience of using pet robots, many compared them to dolls and plush toys, expressing that pet robots would have similar or more impacts on residents since there is an added element of interactivity*: “I’ve seen over the years, residents especially those with dementia, forming a bond with dolls and the teddies.. if the teddy talks or moves she’d (resident) be over the moon [OL2]”.* Compared to live animals, pet robots were thought to be more manageable for residents with dementia, since live animals may have more unpredictable behaviours. From an organisational perspective, pet robots also represented a more hygienic, safer and resource-efficient way forward:

*“Live ducks and hens were introduced in a county home.. it was great for the patients to go out and take in the egg.. staff went on courses to look after these hens and ducks, that only introduced more work.. three residents went to pick up the hen eggs and they fell.. Whereas to me the robots there is no maintaining [HCP7]”.*

Nevertheless, a few preferred live animals, describing tangibility that cannot be replaced with robots: *“It’s the living, breathing, the meows.. whereas this is not real [OL4]”*. Some doubted the impacts or sustained interest over time, as some residents became disinterested or lost interest in interventions such as doll therapy. Therefore, stronger supporting evidence was thought to be necessary to facilitate greater implementation. This should involve evaluating residents’ responses, the proportion of receptive residents, and sustained interest over time.

### Domain 2: Outer Setting

This domain focuses on determinants relating to external influences on implementation, such as external policies and networks with other organisations. Obtaining government funding for pet robots was described by most as difficult, especially for PARO. For public organisations, public funding such as donations, supported the purchase of resources for residents, including the JfA cat. Participants from privately run nursing homes described such sources of funding to be less accessible, as the public would often perceive such organisations as businesses that are focused on profitability, and are therefore less likely to donate funds to them: *“most private nursing homes have a bad name, they will say, well for you it’s a business right? [OL2]*”. The Health Information and Quality Authority (HIQA), a regulatory authority for health and social care standards, was described as having strong influence on care processes. Since pet robots were described as an additional form of activity for residents and could support person-centred care, participants felt that they were well aligned with HIQA’s guidelines and their endorsement of activity provision: *“They were very pro activity provision.. certainly when they discovered that we would have them they would be happy, because it’s person-centred [HCP5]”*. Nevertheless, some expressed concerns about meeting their infection control mandates, because their decisions could have a significant impact on the implementation of pet robots. For example, one participant expressed: *“if they said no that’s it, it’s gone [HCP7]”*. This was especially in light of Covid-19, where infection prevention and control was described by nearly all participants to be paramount. Another participant who had a pet robot within her nursing home shared that all staff were mindful that it was only used with one resident with dementia, and cannot be shared with other residents to prevent cross contamination: *“Even with our experience with the robot there, it’s just for (the resident). Nobody else is touching it and we’ve to be very conscious [OL9].*

With the exception of participants from one of the nursing homes that was a part of a wider group of nursing homes that shared information with each other, others often described minimal networking with other organisations. This was especially pertinent for private homes which typically worked in silo: *“unless they’re a part of a group, generally don’t have a tendency to talk to each other, but kind of they are a business on their own [OL4]”*. However, some expressed interest in knowing other organisations’ experiences with robots, which they felt would influence the implementation of pet robots in their own setting: *“Do they have it in the UK?. We have to probably learn from their experience and their mistakes or positive things [OL2]"*. Nevertheless, a participant from a private nursing home shared that she leveraged on the social media page of Nursing Homes Ireland (NHI), a representative body for nursing homes, which provided some form of networking, as their social media page involved the sharing of other nursing homes’ initiatives.

### Domain 3: Inner Setting

This domain describes determinants relating to the features of nursing homes, such as residents’ needs and resources, the compatibility of robots with existing care processes and workflows, and the availability of resources.

Most participants shared similar sentiments regarding residents’ needs and resources, expressing that residents sometimes felt anxious, lonely, unsettled and were at risk of being passive recipients of care. Most residents had past experiences with animals, but lose access to their pet(s) upon admission. However, *“just because somebody comes into a nursing home does not mean that they stop liking cats or dogs [HCP4]”*. Correspondingly, many (with and without experience of using robots), echoed similar thoughts that implementation was, or would be facilitated, when robots addressed these needs. Like pets, many felt pet robots should be individualised, and should not be shared among residents*.* Participants who had used pet robots echoed similar sentiments, expressing that residents are often reluctant to share pet robots with other residents: *“she won’t let go (of the JfA cat) to anybody else, so they are trying to get more (robots) [HCP11]”.* Nevertheless, residents were described to have fluctuating interests, needs and reduced functional capacities, which could impact their abilities to engage with pet robots.

Residents’ responses to robots had varying influence on staff caregiving. Some described their potential to support caregiving, since care provision would be easier when residents feel comfortable. Such sentiments were congruent with participants who had used pet robots: *“you can see the difference it made to this lady because if not, she’ll be constantly calling for carers [HCP10]”*. Some used robots to encourage residents to engage in routine care: *“We have difficulty giving him supplements. He doesn’t want to take them. And we’ll say well (name of robot) won’t like it if you don’t take your supplement [HCP9]”.* In such sense, the use of pet robots were synergistic with care provision, which facilitated their routine use within the organisation. Participants from one nursing home also described circumstances where one of their residents became disengaged from care routine due to attachment to the pet robot: *“she was so glued to the (JfA) cat she would not eat… would want to feed it and all that… it had to be taken away from her [HCP9]”.* Nevertheless, these participants shared that they managed this situation through formal and informal discussions, (e.g., during handover meetings), to communicate their thoughts and observations of using the pet robots, which helped them tailor their use with residents.

Participants from all nursing homes shared that individual assessments are conducted for all residents. Therefore, most expressed confidence in identifying residents who liked pets and may benefit from pet robots. Since the planning of activities for residents typically usually took place in advance, and pet robots were described as an extension to existing activities, some participants felt that it would not be difficult to integrate it into existing work processes*.* This was echoed by some participants who had used the JfA cat, who felt it aligned with workflow and resources:

*“That’s the beauty of that. You don’t need extra people to administer that (pet robots).. a very important part of anything introduced into long-term care because it really has to be sustainable. No matter how strong people feel about something or how good something is, if there’s a lot of manpower and time needed, it’s hard to see that through* [HCP5]”.

Furthermore, since most residents spend time in a communal room, most nursing staff expressed that they could readily support residents to use pet robots in such communal spaces as a part of their routine work. However, some participants highlighted challenges or potential challenges of using pet robots in communal spaces, such as jealousy between residents, or having residents who dislike them: *“Some enjoyed the (JfA) cat, then there was one lady though… it annoyed her. We ended up having to sort of take the cat out of the room [HCP5]”.*

Management support and a supportive learning climate was described as being important*.*. However, some organisational leaders and occupational therapists felt a lack of capacity to support implementation due to competing responsibilities. Others expressed the need for more information on how to use and manage pet robots. In terms of the relative priority for pet robots, some participants expressed that pet robots were especially relevant during Covid-19, since visitations to nursing homes were restricted. For example, one participant shared that pet robots were introduced into her nursing home during Covid-19, when volunteers could no longer bring in live animals for animal-assisted therapy. However, a few felt that spending financial resources on PARO, in consideration of its cost, should not be prioritised: *“I understand it’s all technology, but there’s so much more that could be bought with that kind of money, we could put that money towards getting a seven seater car to get them out [HCP3]*”. Others shared similar sentiments, citing many existing interventions, or a smaller proportion of residents with dementia among their resident population to benefit from robots.

### Domain 4: Characteristics of Individuals

This domain describes determinants related to individuals involved in the implementation, such as self-efficacy, knowledge and beliefs. Most participants reported that pet robots had a place in dementia care within nursing homes, and believed that technology will be increasingly used to support caregiving. They believed that residents’ needs are evolving, and newer generations of older adults would be more attuned to using pet robots:

*“Years ago, it was mass and it was prayers. That’s out the window. The teddy bear and the pet robot, this all does mean something to them (residents)” [HCP8]*.

Many participants shared beliefs that residents deserve quality of life, and all staff would be supportive of interventions that can benefit residents: *“At the end of the day it’s all about supporting them. When you come into a nursing home, you’re on your end of life journey, you’re basically living in the end. If it (pet robot) makes that journey better, absolutely [OL3]”.* Furthermore, staff derived satisfaction from residents’ joy from interacting with the robots: *“They love it… when you see them laughing and see them so happy. That means a lot, they’re here to live, not here just to be here [HCP7]”*. One participant reported initial scepticism when doll therapy was first introduced within her organisation. After discussions and seeing the impact of dolls on residents, staff grew to be accepting of them. By the time pet robots were introduced, staff showed similar support. The participant also described shared principles of going with residents’ reality in facilitating the adoption of pet robots as a part of routine dementia care:

*“As time has gone by, we’ve come to realise that it’s how that person sees that cuddly robot, that’s what matters. We adjust to their reality now. If this gentleman thinks that it’s a real dog we go with that, rather than trying to bring him into our reality [HCP5]”*.

On the other hand, a participant emphasised the need to use them with residents who could distinguish them as robots: *“you don’t actually want somebody associating with it as if it was a real animal, it could cause further distress down the line if they feel ‘well I’ve never seen it eating’ [HCP10]”*. However, some expressed confidence in managing such situations, such as residents’ attachment to pet robots: *“You’d have some other thing up your sleeve.. You know them so well that you’d know how to deal with a situation.. would be second nature sort of thing [HCP8]"*. Nevertheless, a few were uncertain or ambivalent of their place in nursing homes, felt they suited children or expressed preferences for live pets.

### Domain 5: Implementation Process

This domain describes determinants related to strategies for implementing pet robots, such as planning and engaging stakeholders. Participants identified key stakeholders who are, or should be involved in the implementation of robots. This included activity coordinators, nurses, healthcare assistants, management staff, occupational therapists, residents and family members. Discussion and information sessions were described as necessary for stakeholder buy-in. The implementation process should include an assessment of residents’ preferences for animals, interests, and risk of distress as a part of tailored, person centred care*.* As family members are typically involved in care planning for residents, participants felt it would not be difficult to involve them. In fact, participants shared that family members could support the implementation of pet robots by advising on how to tailor their use for residents. A functional assessment of residents’ cognition, communication, sensory and motor skills was described by occupational therapists as being necessary:

*“Whether somebody has sufficient fine and gross motor skills, whether they’re mobile, can they verbalise their needs. You’d want to be careful that something that’s 2.5KG (PARO) doesn’t end up being a restraint inadvertently… In line with the service provided to residents and our duty of care, we’d probably feel better that it would be assessed [HCP10]”*.

This would guide justifications for use, expected outcomes, and usage indications: *“It’s around the assessment for them and the prescription for the length of time. Because they can get overstimulated by a sensory modulation strategy as well and it can actually lead then to more agitation.. it’s around knowing how best to use it [OL10]”.* Participants suggested that there should be a designated person-in-charge of the robots, responsible for ensuring that their cleaning, maintenance, storage and usage are upkept. Nevertheless, all staff should know how to use the robots, since different staff may be involved in the care of residents each day: *“it could potentially be a bit of a barrier if nobody really knows what’s happening [HCP2].* Participants who had used robots reported that staff would share observations and feedback with each other, discussing ways to manage situations. This need for ongoing review was also raised by other participants, who expressed that it is necessary to consider that residents’ needs, ability and preferences may change over time, and this can affect the appropriateness of pet robots for residents over time*: “people's cognitive function can change over time. And the robot may not be appropriate, it might end up in the back of a press and never taken out again.. I think (a regular review) should be factored into the service of the nursing home” [OL1].*

### Discussion and Implications

To the best of our knowledge, this is the first study to explore multilevel determinants to implementing pet robots in nursing homes for dementia care. Although we anticipated participants to not have prior experiences of using pet robots, some had used them in practice. The determinants described by both groups of participants were generally congruent, although there were some differences in the ‘inner setting’ and the ‘characteristics of individuals’ domains. The cost of pet robots, particularly in relation to PARO, was described as a barrier in relation to other contextual considerations. Participants appeared to conceptualise evidence on pet robots based on non-empirical evidence sources. Although participants (especially those without experiences of using pet robots) expressed desires to learn about other organisations’ experiences, most nursing homes appeared to be working in silos. While the interactivity of pet robots are described as important for engaging residents, participants felt that the interactive features should be balanced with overall affordability. Sentiments on available resources, knowledge and information differed, likely due to different organisational processes, interdisciplinary differences or personal experiences of using pet robots. Despite professional differences, residents’ wellbeing was described as a central priority for all participants. Nevertheless, participants had different beliefs about how pet robots should be used with residents. Overall, determinants within all five domains of the CFIR were interrelated—these interrelations will be further discussed below.

Cost was described as a highly salient determinant. Like several studies involving PARO [11], our participants cited cost as a significant barrier. They further elaborated on this in relation to several individual, organisational and external contextual considerations, such as residents’ needs and resources, internal and external infection control mandates, funding and financial constraints. Furthermore, participants perceived insufficient evidence on its impacts on residents, especially for longer-term engagement. Nevertheless, their expectations of robots appeared to be mediated in relation to the JfA cat, likely due to markedly lower cost. Economic accessibility to the different pet robots therefore highlights a pertinent gap between research and real-world needs [12]. While there is research evidence to support the use of pet robots especially PARO [9, 10], there is a lesser volume of empirical evidence to support the use of the JfA cat [14, 37]. However the lack of knowledge on their empirical evidence did not appear to have a negative impact on participants’ perceptions of their evidence strength, as they referred to evidence from other sources—such as personal experiences with pet robots, an understanding of residents’ unmet needs, and the intervention or supplier source (website for medical supplies). These non-empirical evidence sources facilitated the adoption of the JfA cat as part of their routine work in nursing homes. While participants also expressed the desire for external evidence, such as access to findings from trials or experiences of other nursing homes, there appears to be minimal networking between organisations, which could explain differing levels of implementation. Participants’ description of residents’ unmet needs—such as loneliness, anxiety, and reduced functional capacities—resonated with synthesised findings on the self-reported needs and experiences of nursing home residents with dementia [38].

Like other studies, where PARO better supported residents’ engagement compared to a (non-interactive) alternative [39] our participants also described the realisticness and interactive features of robots as important. However, some doubted the need for advanced interactive abilities, especially if this significantly increase costs. This resonates with a cost-effectiveness study showing that using plush toys were marginally greater value for money than PARO in improving agitation among residents with dementia in care homes [40]. While interactive, lower-cost options such as the JfA pets and Tombot (robot dog) are emerging as potentially more cost-effective options for dementia care, there have been no previous studies comparing robots with different interactive abilities. Future studies are needed to address this gap.

Many participants expressed that robots had addressed, or had the potential to address residents’ needs. Their considerations also entailed residents’ previous occupational roles as pet owners and lovers. As many Irish older adults had experiences with pets, having a pet robot in the nursing home was somewhat synonymous with a ‘typical Irish home’, which was thought as a culturally relevant way of enhancing the familiarity of the environment for residents with dementia [41]. To meet these needs, participants emphasised that residents’ individuality should be respected by considering their design preferences and abilities. In other words, as with other interventions [42], residents should be given the opportunity to uphold their values by choosing a robot that best resonates with them. In terms of product development, developers should also place more emphasis on designing robots to meet these needs. Participants’ description of residents’ needs were often accompanied by mentions of HIQA’s influence on organisational activity provision and person-centred care. Some mentioned about disincentives related to the non-compliance to HIQA’s standards, suggesting that their mandate on infection prevention and control is an important implementation determinant.

Participants had differing sentiments on available resources, knowledge and information, which could be attributed to different organisational processes and structures, disciplinary skillsets and responsibilities. Many organisational leaders, nurses and activity coordinators described “slack resources” [43] within their workflow—such as dedicated time for activities and admission assessments—would enable/has enabled them to ‘squeeze time’ to incorporate pet robots into their work routine. However, opinions on the need for more information on the management and use of robots were varied. Some organisational leaders and OTs emphasised that more comprehensive assessment and re-assessments are needed to ascertain residents’ suitability and need for pet robots, and to design or prescribe individualised intervention plans. Congruent with previous research [12, 44], some participants saw this as necessary to minimise risk of distress from issues such as capacity changes or overstimulation. Nevertheless, allied health professionals highlighted significant manpower and time constraints to support implementation, due to staff shortages. For instance, not all organisations in our study had occupational therapy services. This can inform implementation planning, such as strategic involvement of different stakeholders in different implementation phases, to best leverage different skillsets and resources [45]. Participants agreed that intervention sustainability can be compromised if it demands additional manpower and time. Correspondingly, participants with experience of using robots described their support on caregiving for residents with dementia as facilitators. Ironically, there is a scarcity of studies evaluating the impact of pet robots on caregiving and care processes. Future studies on robots could consider conducting a process evaluation and include these as points of evaluation. Some participants including organisational leaders, perceived a low priority for implementing pet robots, citing reasons such as an existing number of interventions for residents, or a small proportion of residents with dementia. This suggests that apart from considering residents’ needs, organisational needs and workflows should also be considered at the outset.

Despite professional differences, all participants described residents’ wellbeing as a central priority. Therefore, although some staff were initially sceptical or ambivalent about using robots, their attitudes changed after observing their impacts on residents. This finding is supported in the literature [19], suggesting that real-world experiences of using pet robots and evidence from clinical and patient experiences, are necessary to facilitate their uptake. While a few emphasised the importance of ensuring residents’ awareness that robots are not real, most reported comfort with using it with residents regardless of their ability to distinguish it from a live pet. While the ethical issue of ‘deception’ has been critiqued in the literature on pet robots [46], such concerns did not appear to manifest as strongly in practice. This suggests a gap between philosophical ideals and their application to clinical needs and practices. In fact, ethical arguments in the literature appear to be shifting towards acknowledging deception and weighing their impacts on users [47]. This aligns with participants’ explanation of entering residents’ reality, where they supported residents’ belief of robots as live animals, with intentions to support their care. This is similar to the concept of “therapeutic lying”, which is underpinned by principles of empathy, compassion, knowing the person; and is performed to mitigate distress in people with dementia [48]. Similar to existing ethical arguments [46], some participants who did not have experiences with pet robots had concerns that residents may have negative reactions or become attached. However, those with experiences has different views, and reported confidence in managing such situations through professional experiences and discussions with colleagues. This highlights the importance of joint discussion and actions by all key stakeholders to facilitate the adoption of robots in clinical practice.

### Limitations

Like other qualitative studies, there is a likelihood of response bias, where participants may be reluctant to share barriers, especially about their own organisations. Although we aimed to be inclusive and remained responsive to emerging data during analysis, using the CFIR a priori may have led to the exclusion of other determinants. Although some participants had seen or used a pet robot, some had not and based their reporting on a video (i.e., not from actual experiences of use). Nevertheless, the determinants reported by participants with and without experiences were largely congruent, suggesting that anticipated determinants were similar to the actual ones. Determinants of implementation may vary across different countries, where organisations may be governed by different contextual factors. Yet, our study was built upon known domains of implementation, and our findings resonate with findings from international literature. As such, they provide a good general overview of the determinants of implementing pet robots for nursing homes for dementia care.

## Conclusions

In this study, we explored and identified determinants that manifested within all five domains of the CFIR, from the perspectives of organisational leaders and healthcare professionals in nursing homes. The contribution of this study is twofold: it addresses a pertinent knowledge gap in the field of pet robots in the context of dementia care in nursing homes, where little attention has been paid to gain a comprehensive understanding of factors that can impede or enable the implementation of pet robots in real-world practice. Interrelations between determinants clearly highlight that determinants do not occur in silos, and a thorough understanding of multilevel factors should be considered when ascertaining the implementability of pet robots in nursing homes for dementia care. Incongruences between different determinants were also highlighted. For instance, while learning about other organisations’ experiences of pet robots was described as supporting evidence to facilitate the use of pet robots with residents with dementia (CFIR Domain: Characteristics of pet robots), most nursing homes in the study described minimal networks with other organisations (CFIR Domain: Outer setting). Secondly, these findings are of practical utility for researchers and stakeholders from nursing homes, as they provide a springboard for identifying and designing contextually relevant implementation strategies to guide the translation of pet robots from research into real-world practice.

## Supplementary Information

**Additional File 1.** 

**Additional File 2.** 

**Additional File 3.** 

**Additional File 4.** 

**Additional File 5.** 

## Data Availability

The framework that was developed and applied to the dataset is available as a supplementary file (Additional File 4). The data generated and analysed in this study is not publicly available in order to maintain participant privacy and confidentiality. However, de-identified parts of the interview transcripts may be obtained from the corresponding author upon reasonable request.

## Abbreviations

CFIR: Consolidated Framework of Implementation Research
HCP: Healthcare professionals
OL: Organisational leaders
