# Supplementary material for: Determinants of implementing of pet robots in nursing homes for dementia care
Source: BMC Geriatr. 2022 May 27;22:457. doi: 10.1186/s12877-022-03150-z (PMC9136197; doi:10.1186/s12877-022-03150-z)
Supplement: Supplementary file 1 — Additional File 1. [file 12877_2022_3150_MOESM1_ESM.docx]

# **Additional File 1 – Summary of PPI meetings**

| **Date** | **Mode** | **Duration** | **Agenda** |
| --- | --- | --- | --- |
| 13 Jul 2021 | Meeting | 1H | Explained about project to PPI member, discussed about potential PPI input in this research and project timelines |
| 13 Jul 2021 | Email  (pre-meeting preparation) | 0.5H | Emailed interview guide to PPI member one week in advance in preparation for the next meeting |
| 19 Jul 2021 | Meeting | 1H | Discussed and sought feedback on interview guide for PLWD |
| 5 Aug 2021 | Meeting | 1H | Showed video of pet robots to PPI member and sought feedback on the video |
| 13 Sep 2021 | Meeting | 1H | Discussed about difficulties recruiting people with dementia as participants and discussed other potential recruitment platforms |
| 21 Jan 2022 | Email  (pre-meeting preparation) | 0.5H | Emailed document on a summary of qualitative study findings to prepare for next meeting |
| 26 Jan 2022 | Meeting | 1H | Discussed about data from qualitative study and interpretation |
