# Supplementary material for: Determinants of implementing of pet robots in nursing homes for dementia care
Source: BMC Geriatr. 2022 May 27;22:457. doi: 10.1186/s12877-022-03150-z (PMC9136197; doi:10.1186/s12877-022-03150-z)
Supplement: Supplementary file 2 — Additional File 2. [file 12877_2022_3150_MOESM2_ESM.docx]

# **Additional File 2: Video content**

***Video content***

- Self-Introduction
- Introduction to PARO
  - Demonstrate how PARO can be switched on/off
  - Soft, white fur covering
  - Sensors (touch sensors, temperature sensors, position sensors, voice recognition)
  - Movements (making sounds, moving its flippers, looking at the user)
  - Ability to learn behaviours that the user enjoys and develop a personality
  - Length and weight
  - Battery duration, charging and charging duration
  - Cost of PARO and warranty
- Introduction to the Joy for All cat
  - Demonstrate how the Joy for All cat can be switched on/off
  - Sensors (touch sensors, light sensors
  - Movements (purring, vibration, meowing, body movements, eye movements)
  - Not able to learn behaviours or develop a personality
  - Length and weight
  - Battery duration, battery type and compartment
  - Cost of the Joy for All cat and warranty
- Overall care of the pet robots
  - Ways that the pet robots can be damaged
    (holding them too tightly or dropping them on the floor)
  - Non-waterproof and cleaning using wipes and sprays

Total length of the video: 05:48 minutes

Narrator: Wei Qi Koh

***Acknowledgements:***
We are grateful to Mr Fergus Timmons and Ms Mary Higgins from Alzheimer Society Ireland (ASI) for the loan of PARO and the Joy for All cat.
