# Supplementary material for: Determinants of implementing of pet robots in nursing homes for dementia care
Source: BMC Geriatr. 2022 May 27;22:457. doi: 10.1186/s12877-022-03150-z (PMC9136197; doi:10.1186/s12877-022-03150-z)
Supplement: Supplementary file 3 — Additional File 3. [file 12877_2022_3150_MOESM3_ESM.docx]

**Additional File 1 - Interview Guide**

1. What do you think about the two pet robots that you saw in the video?
2. Would you want to introduce pet robots into your workplace for residents with dementia? If yes, why (and if no, why not?)
3. What are some local or national policies/guidelines, if any, that has influenced dementia care in your workplace?
4. Do you know of other nursing homes of care organisations that have introduced pet robots for dementia care?
5. How do you feel about using pet robots as a part of your day-to-day work with residents with dementia?
6. What do you think would be required to introduce pet robots for residents with dementia in your workplace?
