# Supplementary material for: Determinants of implementing of pet robots in nursing homes for dementia care
Source: BMC Geriatr. 2022 May 27;22:457. doi: 10.1186/s12877-022-03150-z (PMC9136197; doi:10.1186/s12877-022-03150-z)
Supplement: Supplementary file 4 — Additional File 4. [file 12877_2022_3150_MOESM4_ESM.docx]

Nodes\\Phase 3 - Indexing

| CFIR domain / node / subnode | Description |
| --- | --- |
| 1. Intervention characteristics |  |
| Adaptability | Includes statements pertaining to the extent to which/how the use of pet robots can or should be adapted, tailored, refined or reinvented to meet the local needs of residents with dementia in the nursing home. Exclude statements relating to the design (e.g. suggestions that the pet robot should be designed as a dog or another animal) that people are more familiar with, and code it to "Design quality and packaging - Familiarity" |
| Complexity | Includes statements pertaining to the perceived difficulty of using pet robots, as reflected by the scope, intricacy, number of steps or duration needed to use pet robots. This includes all elements of using pet robots including the manner in which they are charged. |
| Cost | Includes statements pertaining to the cost of pet robots and costs associated with implementing pet robots, including investment, supply, opportunity or maintenance costs |
| Design quality and packaging | Include generic statements regarding the perceived excellence in how pet robots are bundled, presented and assembled (design quality and their packaging) that do not fit in any of the sub codes below Exclude statements that can be captured in the sub-codes below, and code it to the relevant subcode |
| Charging | Include statements/descriptions about how the pet robots are being charged. Exclude statments if the descriptions are in relation to the complexity of charging (e.g., ease of use), and code to "complexity" |
| Familiarity (cultural relevance) | Include statements about the familiarity or unfamiliarity of PARO or the JfA cat's design (as a seal/cat), and comments about the cultural relevance of the design to residents and staff. Also include comments relating to alternative designs (other animals) which may be more familiar or culturally relevant |
| Physical, interactive and tactile features | Includes statements relating to the physical appearance, interactive & tactile features, for example their eyes, fur, pacifier, realistic looking, colour, weight, interactive functions (e.g. the movement, sounds, programming, artificially intelligence), softness, sensory element Exclude statements describing the familiarity of the design of pet robots (e.g. whether they are familiar or unfamiliar to residents/staff) and its cultural relevance, and code it to subcode “Familiarity (cultural relevance)” |
| Evidence strength and quality | Include statements regarding awareness of evidence and the strength and quality of evidence supporting the use of pet robots, as well as the absence of evidence or a desire for different types of evidence instead of evidence from the literature. May also include comments that more evidence is required to support their use in the nursing home for dementia care. Exclude statements regarding the need for evidence for funders (funding bodies), and code it to "External policy and incentives - external funding |
| Intervention source | Includes statements about pereceptions about whether the pet robots were developed externally (outside their organisation) or developed internally (developed by their nursing home) |
| Relative advantage | Includes statements about the advantages or disadvantages of using pet robots as compared to other interventions (e.g. live animals, doll therapy, music, bingo). Excludes statements of an individual’s personal experience with interventions similar to pet robots (e.g. doll therapy, plush toys, technological devices) to support dementia care if the individual does not specifically compare it to pet robots, and code to "Knowledge and beliefs - Personal experience with using similar interventions" |
| Trialability | Includes statements that a trial / testing pet robots on a small scale in the nursing home was/would be welcome and possible, or that it is is possible to reverse course (de-implement) if needed |
| 2. Outer setting |  |
| Cosmopolitanism | Includes statements about how the nursing home is networked with other external organisations/nursing homes, or descriptions of outside group memberships and networking between the nursing home and other care setting/organisations |
| External funding bodies | Includes statements or comments about the external funding bodies that can directly influence the funding that is necessary to support the introduction of pet robots (e.g. HSE, public funding, charity organisations, fund raising etc) |
| External policies and incentives | Include statements about the external strategies (national or wider policies, regulations, and guidelines) outside the nursing home that has influenced or could influence the introduction/implementation of pet robots for dementia care Excludes statements relating to external funding and code it to “external funding bodies” |
| Peer pressure | Include statements about perceived pressure or motivation from other entities or organisations within the county or country to implement pet robots (i.e., mimetic or competitive pressure to implement pet robots, because most or other key peer or compeititve organisations have already implemented or are in a bid for a competitive edge) |
| Residents' needs and resources | Include general statements demonstrating the individual’s awareness/lack of awareness of the needs and resources of residents with dementia that cannot be coded into any of the subcodes below. This could include statements about residents’ preferences or needs for pet robots, or the challenges/facilitators that residents could experience while living in the nursing home or when using pet robots. Exclude statements that can be coded into the subcodes below, and code it to the respective subcodes |
| Demeaning or infantilisation | Includes statements that pet robots would be perceived by residents as something demeaning/infantilising/embarassing |
| Fluctuating interests | Statements mentioning residents' interest/disinterests in pet robots or other similar interventions, and possible changes to these levels of interest over time |
| Needs of residents with dementia | Comments related to residents' desire to nurture, take care, or show love and compassion, multisensory needs, need for companionship, need for individual pets etc |
| Cognitive, spiritual and psychosocial needs | Includes statements that the pet robots may (or may not) meet the cognitive, spiritual and psychosocial needs of residents with dementia in the nursing home |
| Physical needs | Includes statements that the pet robots may (or may not) meet the physical needs of residents with dementia in the nursing home |
| Past experiences with animals | Include statements about residents' past experiences with animals or pets (e.g. have had animals/pets previously, liked animals/pets etc) |
| 3. Inner setting |  |
| Culture | Include statements related to the values that the nursing home stands by, and the culture, norms or assumptions that are assumed within the nursing home |
| Implementation climate | Include statements regarding the implementation climate (general level of receptivity to implementing pet robots) that do not fit into any of the below subcodes. Exclude statements that can align with and can be coded to the subcodes, and code it to the relevant subnode. |
| Compatibility | This code describes the degree of tangible fit between how using pet robots aligns with/does not align with the existing work flows and systems in the nursing home. Code the relevant codes into the relevant subcodes below (based on their description). |
| Care process for residents | Include statements that demonstrate the level of compatibility the social robot has with the work processes of providing care for residents with dementia. This may also include statements about whether using pet robots is/would be an added work load to the current care processes for residents |
| Infection control and cleaning practices | Comments related to use of pet robots in relation to infection prevention and control practices within the nursing home (e,g, comments about possible cross contamination related to the use of pet robots, added/reduced infection control/cleaning work if pet robots were used) |
| Intervention strategy | Includes statements about how pet robots are intended to be used/are used with residents with dementia (e.g. individual interventions, group interventions) within the nursing home |
| Support care workers | Include statements describing how the pet robots are/can complement or be used to support the work of staff in the nursing home |
| Goals and feedback | Includes statements about how the nursing home’s goals of using pet robots are/should be communicated to and fed back to staff (e.g., comments that audits/regular feedback about within the nursing home is necessary to support the use of pet robots to meet the goals of the nursing home, or comments about such audits/feedback) |
| Learning climate | Include statements that support (or refute) the degree to which the nursing home exhibits a ‘learning climate', in which: 1) leaders express their own fallibility and need for team members’ assistance and input; 2) team members feel that they are essential, valued, and knowledgeable partners in the change process; 3) individuals feel psychologically safe to try new methods; and d) there is sufficient time and space for reflective thinking and evaluation. |
| Organisational incentives and rewards | Includes statements about incentives/rewards that the nursing home can provide/has provided to support the use of pet robots.Tangible incentives/rewards can include such as goal-sharing awards, performance reviews, promotions and raisies in salary; and less tangible incentives may include increased stature or respect |
| Relative priority | Includes statements about the importance/relative priority of implementing pet robots within the nursing home (E.g. comments comparing the priority of introducing pet robots in light of to other ongoing intervention, or priority of introducing pet robots in light of situations such as Covid-19) Exclude statements regarding the priority of the innovation based on compatibility with organizational processes/values and code it to relevant subnode in “Compatibility” (e.g., if an innovation is not prioritized |
| Tension for change | Includes statements that demonstrate a strong need for pet robots or that the current situation in the nursing home is untenable (e.g. comments that pet robots are absolutely necessary or absolutely redundant) Exclude statements that demonstrate the innovation is better (or worse) than existing programs and code to Relative Advantage. |
| Networks and communications | Include statements about general (formal and informal) networking, communication, and relationships within the organisation/setting, and statements related to team formation, quality, and functioning |
| Readiness for implementation | Include statements regarding the general level of readiness for implementation. Exclude statements regarding the general level of readiness for implementation that are captured in the sub-codes below. |
| Access to knowledge and information | Include statements related to the individual's access to access to knowledge & information about interventions including pet robots. This can come from internal (within the nursing home) or external sources - e.g. experts, other staff, training etc Exclude statements related to engagement strategies and outcomes (e.g. how the individual became engaged with the innovation) and code to “Engaging: key stakeholders”.Exclude statements about general netwoking and code to “Networks and communications" |
| Additional knowledge and support | Include statements that stakeholders require access to additional knowledge and information to support the use of pet robots. These can Include knowledge and information that are not currently available from within the nursing home (e.g. staff training) or those available from external entities/organisations |
| Additional resources required | Includes statements about the additional resources that are required in order to introduce pet robots into the nursing home for residents with dementia. This might include money, training, education, physical space, and time. Exclude statements relating to the need for external funding, and code it to outer setting – “External funding bodies” |
| Available resources | Includes statements about the resources that are available to introduce pet robots into the nursing home for residents with dementia. This might include money, training, education, physical space, and time. Exclude statements relating to the need for external funding, and code it to outer setting – “External funding bodies” |
| Leadership engagement | Include statements regarding the level of commitment/support of leadership towards the implementation of the social robot. |
| 4. Characteristics of individuals |  |
| Individual stage of change | Includes statements about the phase of change that the individual is in (pre-contemplation, contemplation, preparation, action and maintenance). This can include a range of comments about the stages of change - e.g. the individual is comfortable with using pet robots (e.g. maintenance), or is adverse to the idea of using pet robots (precontemplation) |
| Knowledge and beliefs about the intervention | Includes general statements about the individual’s attitudes and values placed on pet robots or similar interventions (e.g. technology in dementia care, doll therapy) that cannot be coded into any of the subnodes below. Exclude statements that can be coded into any of the subnodes below, and code to the relevant subnodes |
| Perceptions for future use of technology | Include statements mentioning the individual's general belief or attitudes about the future use of technology (including pet robots) in dementia care and/or in nursing homes (i.e., Perceptions of technology in general in dementia care) |
| Personal experience with using similar interventions | Include statements of personal experience in interventions similar to pet robots (e.g. doll therapy, plush toys, technological devices) to support dementia care for PLWD. Exclude statements that directly compare these interventions with pet robots in terms of their relative advantages/disadvantages, and code to "Relative advantage" |
| Treating the robot like it is real | Includes statements about the individual's thoughts/perceptions if a resident with dementia were to treat a pet robot as if it was a real animal. Exclude statements if participant provided context specific information/examples from their nursing home (e.g. similar circumstances arising from the use of dolls, etc) and mention that such scenarios may be applied to the use of pet robots, and code to "Compatibility - Care process" |
| Other personal attributes | Include statements that include other personal traits such as tolerance of ambiguity, intellectual ability, motivation, values, competence, capacity, and learning style. Also includes personal motivation to network with other institutions that would allow them to know what other activities are used to support or enhance dementia care |
| Self efficacy | Include statements related to belief in their own capabilities (confidence in their ability) to execute courses of action to achieve implementation goals |
| 5. Process |  |
| Engaging | Include statements related to engagement strategies and outcomes (i.e. if and how stakeholders became engaged with the social robot and what their role is in implementation). Exclude statements that are captured in the sub-codes below. |
| Champions | Include statements related to how the individuals within the organisation (who identify themselves as champions to support, market, ‘drive through’, or, overcome indifference or resistance) engaged with the implementation of pet robots, Exclude statements regarding leadership engagement, and code to “Leadership Engagement” e.g. if a champion is also an organizational leader, e.g., if a director of primary care takes the lead in implementing pet robots |
| External change agents | Include statements related to external stakeholders’ engagement strategies and outcomes (e.g. how he/she became engaged with pet robots and their role is in implementation (e.g. how they supported implementation efforts). External change agents formally influence or facilitate intervention decisions in a desirable/undesirable direction, and may include researchers and family members etc (who are external to the organisation) Exclude statements regarding facilitating activities, such as training to use pe |
| Formally Appointed Internal Implementation Leaders | Include statements related to how the individuals within the organisation who been formally appointed with responsibility for implementing an intervention (e.g. activity coordinator, recreational therapists) engaged/should engage with the implementation of pet robots, and the outcomes of such engagement |
| Opinion leaders | Include statements related to how the opinon leader engaged/should engage with the implementation of pet robots, and the outcomes of such engagement |
| Residents with dementia | Include statements related to how residents should be engaged/became engaged with introduction or implementation of pet robot within the nursing home, and the outcomes of resident engagement in implementation efforts. Exclude statements demonstrating (lack of) awareness of the needs and resources of those served by the organization and whether or not that awareness influenced the implementation or adaptation of the social robot and code to “Needs and Resources” |
| Staff | Include statements related to how other staff (not mentioned in the other subnodes in the "Engaging" node) should be engaged/became engaged with introduction or implementation of pet robot within the nursing home, and the outcomes of staff engagement in implementation efforts. Staff (can be a group of staff) in an organization who have formal or informal influence on the attitudes and beliefs of their colleagues with respect to implementing the pet robots and similar interventions |
| Executing | Include statements that demonstrate how implementation occurred with respect to the implementation plan. (Note: Executing is coded very infrequently due to a lack of planning. However, some studies have used fidelity measures to assess executing, as an indication of the degree to which implementation was accomplished according to plan). |
| Planning | Include evidence of pre-implementation diagnostic assessments and planning, as well as refinements to the plan, i.e. - the degree to which a scheme or method of behavior and tasks for implementing an intervention (pet robots) are developed in advance, and the quality of those schemes or methods. |
| Pre-assessment | Includes statements about the need for a needs, eligibility or risks assessment of residents' needs (or statements about a similar pre-assessment that is being used) to ensure that the pet robots align to residents' values and abilities, or to ensure that risks associated with the use of pet robots are considered |
| Regular (ongoing) review | Includes statements relating to the need for regular or ongoing review of pet robots as a part of the implementation process to sustain change |
| Reflecting and evaluating | Include statements that refer to the NH’s reflection and evaluation of the process of implementing pet robots, such as the NH’s ongoing review (or lack of ongoing review) of the process of introducing pet robots. Exclude statements related to the (lack of) alignment of the pet robots with organizational goals/feedback to staff, e.g., audit & feedback, and code to “Goals & Feedback”. Exclude statements that capture reflections/evaluation from participants during interview, code to "Knowledge & beliefs" |
